# Conversion Techniques for Immediate-Loading Interim Implant-Supported Complete-Arch Fixed Dental Prostheses (ISCFDPs): Four Clinical Reports

**DOI:** 10.3390/dj14060350

**Published:** 2026-06-08

**Authors:** Toshiki Nagai, Chao-Chieh Yang, Amal Al-Faraj, Matthew G. Thompson, Elizabeth Rubalcava, Apisit Akarapattananukul, Wei-Shao Lin

**Affiliations:** 1 Department of Prosthodontics, School of Dentistry, Indiana University, Indianapolis, IN 46202, USA; 2 Department of Prosthodontics and Dental Implantology, College of Dentistry, King Faisal University, Al Ahsa 31982, Saudi Arabia; 3 Department of Dental Services, Naval Medical Center Portsmouth, Portsmouth, VA 23708, USA

**Keywords:** immediate loading, implant-supported complete-arch fixed dental prosthesis, denture conversion, guided conversion, closed-mouth pickup technique, digital workflow

## Abstract

**Background/Objectives:** Immediate-loading interim implant-supported complete-arch fixed dental prostheses (ISCFDPs) are widely used for immediate loading in edentulous patients. Although traditional denture conversion techniques are well established, newer systems aim to improve efficiency and prosthesis integrity. This clinical report aims to describe and compare four chairside conversion techniques for immediate-loading interim ISCFDPs. **Methods:** Four clinical cases were treated using different conversion techniques, including conventional denture conversion, guided conversion with static computer-assisted implant surgery (s-CAIS), and two closed-mouth pickup systems (SMART Denture Conversion and EasyPro). Clinical workflows, procedural characteristics, and prosthetic considerations were evaluated. **Results:** All techniques enabled the successful fabrication of immediate-loaded interim ISCFDPs. Conventional conversion was flexible and cost-effective but technique-sensitive and associated with increased risk of prosthesis weakening. Guided conversion improved structural integrity and reduced intraoral adjustment but required precise planning and higher costs. Closed-mouth systems preserved occlusion, minimized denture modification, and reduced chairside time, though they relied on proprietary components and had limited clinical evidence. **Conclusions:** Each conversion technique presents distinct advantages and limitations. Selection should be based on clinical conditions, available resources, and clinician experience. Further studies are needed to validate the long-term outcomes of emerging conversion systems.

## 1. Introduction

As the global population ages, the prevalence of edentulism is increasing worldwide. Implant-supported complete-arch fixed dental prostheses (ISCFDPs) offer an efficient solution for rehabilitating fully edentulous jaws [1]. Various loading protocols for ISCFDPs have consistently shown high implant survival rates [2,3]. Consequently, immediate loading with immediate-loading interim ISCFDPs has become a preferred treatment approach for edentulous patients after placing at least four implants [4,5,6]. Traditionally, clinicians convert an interim complete denture into an immediate-loading interim ISCFDP to enable immediate loading [6,7,8,9,10], although several alternative techniques have also been introduced [4,11,12,13,14]. The complete denture conversion technique involves attaching interim cylinders to the implants or abutments, securing them to a complete denture with resins, and modifying the patient’s existing denture into an immediate-loading interim ISCFDP. Creating precise access openings in the denture before the conversion is essential to keep the interim cylinders from displacing the denture; otherwise, extensive modifications to the prosthesis may be required. In addition, ensuring the proper placement of access openings is critical; otherwise, the resultant immediate-loading interim ISCFDP will be weakened and its occlusal surface integrity compromised. The increased amount of pickup material required would exacerbate resin shrinkage and impair the passive fit of the interim ISCFDP [6,7,8,9,10].

The most common prosthetic complication associated with interim ISCFDPs is prosthesis fracture [15,16,17]. To reduce this risk, several strategies have been proposed, including minimizing or eliminating the cantilevered sections [18], incorporating metal frameworks [19,20,21,22], employing milled denture techniques [13,23,24], using static computer-aided implant surgery (s-CAIS) with a prefabricated conversion prosthesis to reduce the size of access holes [24], adopting closed-mouth pickup conversion techniques with specialized separable components [25,26], and leveraging other digital tools, such as photogrammetry, specialized scan bodies with auxiliary features, and laboratory-fabricated 3D-printed prostheses [27]. These methods collectively enhance the strength and stability of the interim ISCFDPs. Although different techniques were adopted for the interim ISCFDPs used for the immediate-loading prosthesis, those that permit chairside conversion remain popular because they improve efficiency, reduce clinical time, and streamline the immediate-loading workflow [24,25,26]. Nevertheless, the converted immediate-loading interim ISCFDPs are still prone to fracture, typically at the pontic midline and in the thinned buccal and lingual walls created when enlarging denture access openings to accommodate interim cylinders [23].

Although numerous chairside conversion techniques have been developed for immediate-loading interim ISCFDPs, comparative information on their requirements, advantages, and disadvantages is still limited. The authors aim to clarify the chairside post-implant-placement conversion methods for interim ISCFDPs by analyzing four clinical case reports. Highlighting the available systems, this review seeks to help clinicians make informed decisions and to improve treatment outcomes.

## 2. Clinical Reports

Prior to treatment, diagnostic dentures were fabricated in all cases to establish the ideal tooth position and vertical dimension of occlusion as part of the prosthetically-driven treatment planning. Immediate-loading interim ISCFDPs were selected based on adequate primary implant stability, favorable occlusal conditions, and the goal of providing an immediate-loading fixed function while avoiding removable prostheses during the healing phase. Additional considerations included patient preference, prosthetic predictability, and preservation of the established occlusal relationships.

### 2.1. Case 1—Conventional Conversion

**Figure 1:**
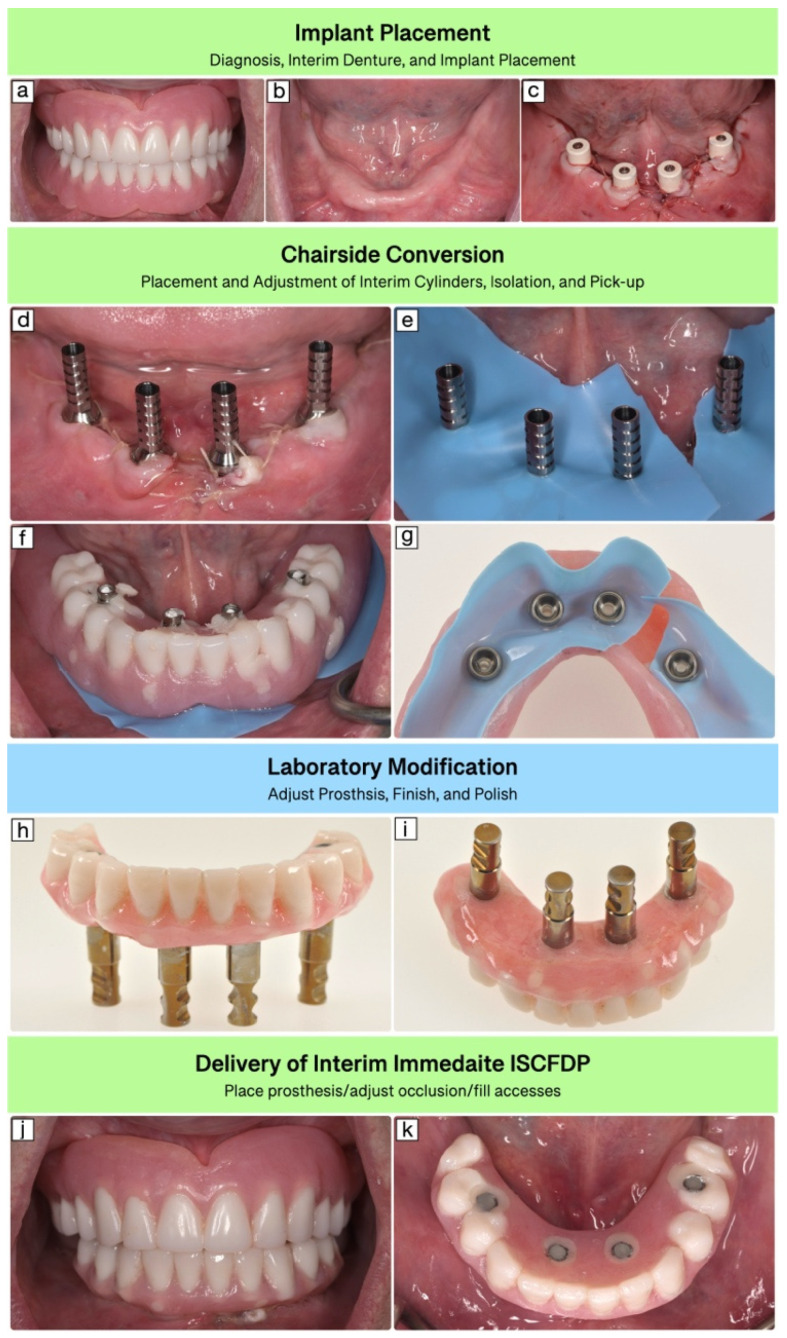
(**a**) Frontal view of the mandibular interim complete denture in situ; (**b**) Occlusal view of the mandibular ridge; (**c**) Post-implant placement; (**d**) Interim cylinders secured on the implant; (**e**) Rubber dam placement; (**f**) Auto-polymerizing acrylic resin used to connect the interim cylinders and denture; (**g**) The denture was removed, and the interim cylinders were inspected to ensure they were all securely connected to the denture with acrylic resin, showing no movement or mobility; (**h**) Finished and polished interim immediate-loading implant-supported complete-arch fixed dental prosthesis (ISCFDP); (**i**) Intaglio surface of the interim immediate-loading ISCFDP; (**j**) Frontal view of the interim immediate-loading ISCFDP; (**k**) Occlusal view.

After prosthetic-driven planning, four mandibular implants were placed with adequate primary stability (insertion torque > 35 Ncm) to permit immediate loading. Multi-unit abutments (e.g., screw-retained abutments, Institut Straumann AG, Basel, Switzerland) were torqued (35 Ncm) per manufacturer recommendations (Figure 1a–c), and non-engaging temporary cylinders were connected. Access channels were prepared in the existing interim denture to accommodate the cylinders and to maintain seating (Figure 1d). A rubber dam barrier and block-out protected undercuts, along with auto-polymerizing acrylic resin were used to lute the cylinders to the denture base (Figure 1e,f).

After polymerization for approximately 9 min, the prosthesis was removed to confirm the secure integration of the abutments (Figure 1g). Any voids were filled with additional PMMA resin, and the provisional restoration was placed in a pressure pot to optimize polymerization at 15 pounds per square inch (PSI) for 5 min. The prosthesis was recontoured to a fixed full-arch interim form with convex intaglio for hygiene, finished/polished, and delivered with light occlusal refinement using prosthetic screws torqued to 15 Ncm (Figure 1h,i). Screw channels were sealed with polytetrafluoroethylene (PTFE) tape and provisional material (Figure 1j,k). This technique leveraged the readily available components and had a familiar workflow but required careful access positioning to avoid prosthesis thinning and subsequent fracture risk (Figure 1). The entire procedure time is about 45–90 min.

### 2.2. Case 2—Guided Conversion

**Figure 2:**
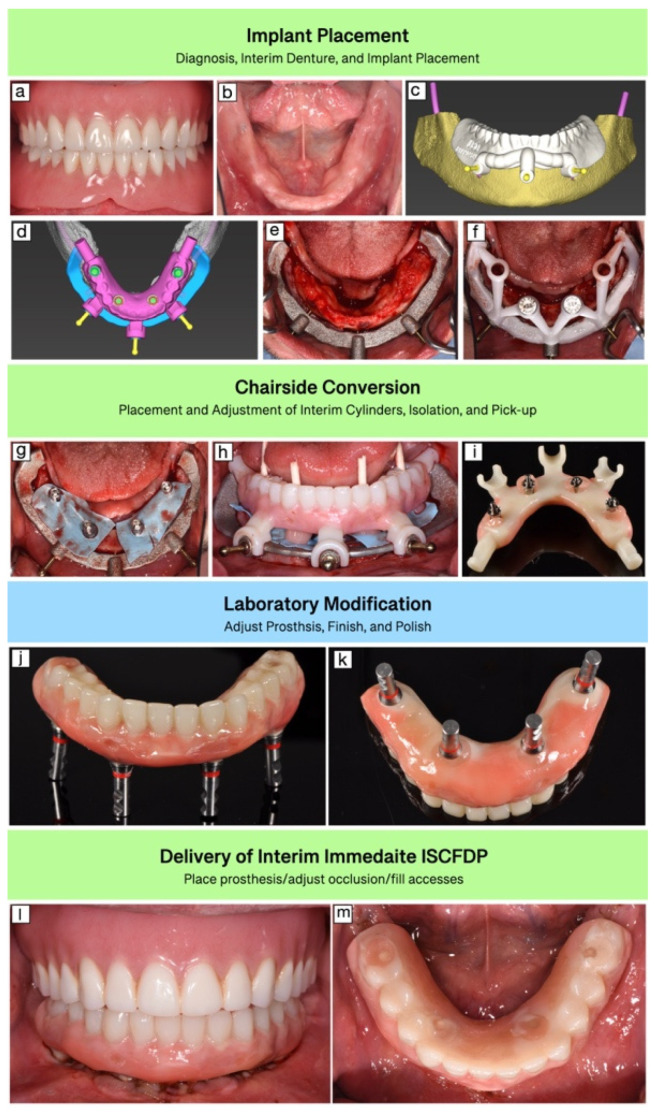
(**a**) Frontal view of the mandibular interim complete denture in situ; (**b**) Occlusal view of the mandibular ridge; (**c**) Digital design of the fixation pin placement template; (**d**) Digital design of the fixation pin-supported bone reduction template and immediate-loading prosthesis; (**e**) Alveolar ridge reduction completed under the guidance of the bone reduction template; (**f**) Implant placement following the fixation pin-supported implant placement template; (**g**) Rubber dam placement; (**h**) Auto-polymerizing resin used to connect the interim cylinders and immediate-loading prosthesis; (**i**) The denture was removed, and the interim cylinders were inspected to ensure they were securely connected to the denture with acrylic resin, showing no movement or mobility; (**j**) Finished and polished interim immediate-loading implant-supported complete-arch fixed dental prosthesis (ISCFDP); (**k**) Intaglio surface of the interim immediate-loading ISCFDP; (**l**) Frontal view of the interim immediate-loading ISCFDP; (**m**) Occlusal view.

Using a dual-scan protocol with cone beam computed tomography (CBCT), static computer-assisted implant surgery (s-CAIS) was planned along with stackable templates (fixation-pin, bone-reduction, and implant-placement guides) and a prefabricated immediate-loading prosthesis (Figure 2a) [22,24]. Following implant placement (insertion torque > 35 Ncm) and abutment connection (35 Ncm) (Figure 2b,c), the prefabricated prosthesis, incorporating the predesigned access channels and vertical stops, was seated on the bone-reduction template to maintain the planned maxillomandibular relationship and to minimize any occlusal adjustments. Temporary cylinders were picked up intraorally with auto-polymerizing acrylic resin (Figure 2d–f).

After polymerization for approximately 9 min, the prosthesis was removed to confirm the secure integration of the abutments (Figure 2g). Any voids were filled with additional PMMA resin, and the provisional restoration was placed in a pressure pot to optimize polymerization at 15 pounds per square inch (PSI) for 5 min. The prosthesis was finished and polished extraorally before delivery using prosthetic screws torqued to 15 Ncm (Figure 2h–k). This protocol reduced the intraoral adjustment and preserved prosthesis integrity, but it was highly dependent on accurate preoperative planning and surgical execution. Increased costs and limited intraoperative flexibility remained important considerations, and deviations from the digital plan could necessitate enlarging access channels or reverting to a conventional conversion (Figure 2). The entire procedure time is about 35–60 min.

### 2.3. Case 3—Closed-Mouth Pickup Conversion—Example 1 (SMART Denture Conversion, Keystone Dental Group)

**Figure 3:**
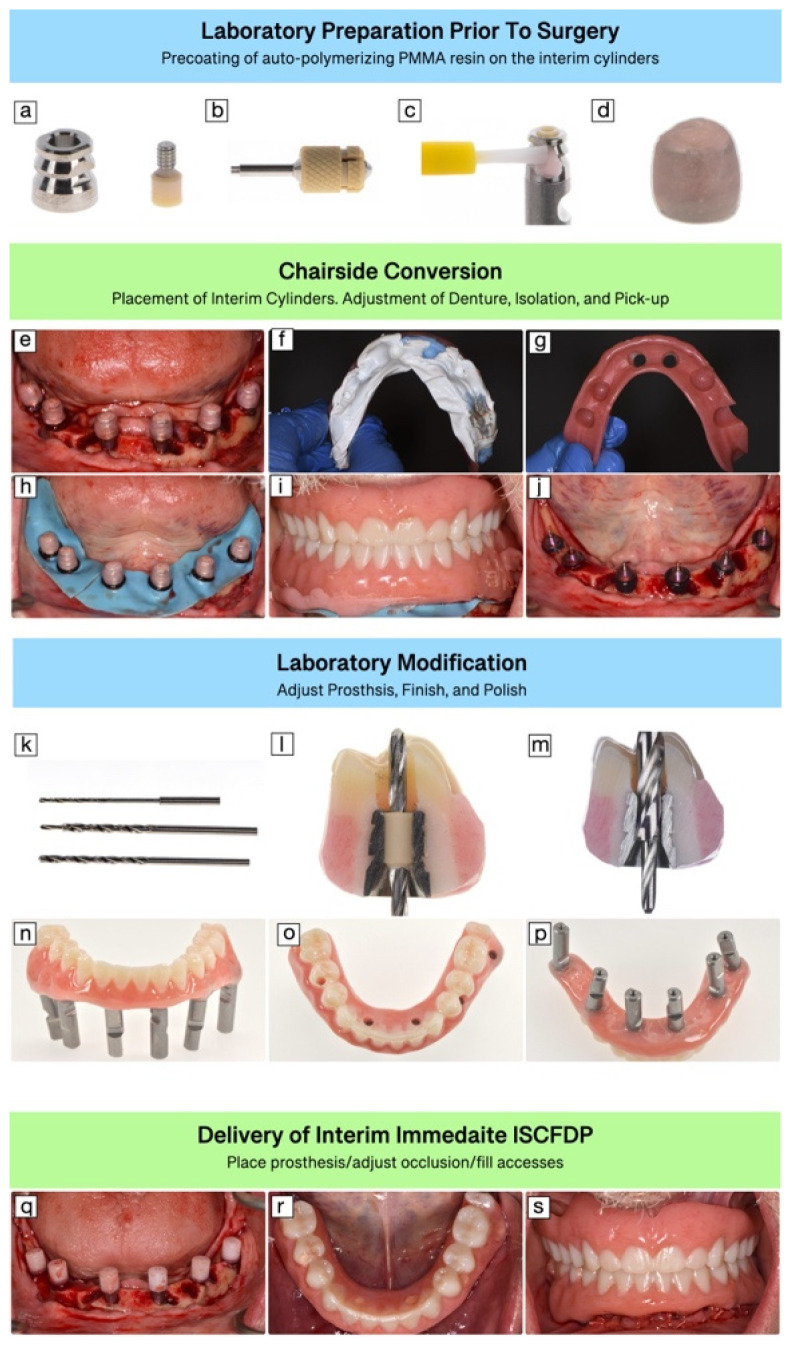
(**a**) Low-profile interim cylinder and separable implant screw, consisting of a polyetheretherketone (PEEK) cap and threaded body; (**b**) Low-torque driver; (**c**) Precoating the resin on the assembled units of interim cylinders with separable implant screws; (**d**) Coated assembled units; (**e**) Precoated assembled units of interim cylinders with separable implant screws were hand tightened on the multi-unit abutments; (**f**) The intaglio of the denture was coated with polyvinyl siloxane and PTFE tape to create indents of the precoated assembled units; (**g**) Relief areas were adequately created. (**h**) Rubber dam and block-out material in situ; (**i**) Patient was guided in the centric occlusion position during the closed-mouth pick-up procedure; (**j**) The prosthesis was removed from the mouth, and the threaded bodies of the separable implant screws remained in the multi-base abutment; (**k**) Three-step proprietary drill system; (**l**) Pilot drill; (**m**) Access drill; (**n**) Finished and polished interim ISCFDP in frontal view; (**o**) Occlusal view; (**p**) Intaglio surface; (**q**) Press-on caps were used to remove the threaded bodies; (**r**) Occlusal view of the interim immediate-loading ISCFDP; (**s**) Frontal view.

This closed-mouth approach used low-profile titanium cylinders and separable screws (polyetheretherketone (PEEK) cap and threaded body) (Figure 3a–d). After implant placement (insertion torque > 35 Ncm) and multi-unit abutment connection (35 Ncm), precoated cylinder–screw assemblies were hand-seated, and the existing denture was relieved and seated in centric occlusion with quick-setting polyvinyl siloxane (PVS) indexing to confirm the locations (Figure 3e–g). With rubber-dam isolation and block-out, acrylic was applied to the intaglio, and the patient maintained centric occlusion (CO) during polymerization, preserving the vertical dimension and occlusal scheme (Figure 3h,i). After approximately 9 min, the prosthesis was removed using vertical dislodging force. Threaded posts remained on the abutments while the PEEK caps/cylinders stayed in the prosthesis (Figure 3j). Any voids were filled with additional PMMA resin, and the provisional restoration was placed in a pressure pot to optimize polymerization at 15 pounds per square inch (PSI) for 5 min. Final screw access channels were created using a three-step proprietary drill system included in the manufacturer’s starter kit (Figure 3k), as follows:

(1) A pilot drill was used from the intaglio surface through the temporary cylinder (Ti base) to the occlusal surface of the prosthesis (Figure 3l).
(2) An access drill was used from the occlusal aspect through the pilot channel to the temporary cylinder (Ti base), with a built-in stop to prevent damage (Figure 3m).
(3) A clearance drill, mounted on a hand tool, was used to remove the PEEK screw heads through the occlusal openings, completing the access preparation.

The interim ISCFDP was then finished and polished to establish proper anatomical contour and surface smoothness for intraoral delivery (Figure 3n–p). Using gentle finger pressure, seat the press-on caps (POCs) (Smart Denture Conversions, Apex, NC) onto each screw-retained abutment (SRA) until they fully engaged the exposed ends of the threaded posts (Figure 3q). Subsequently, carefully remove the press-on caps by an unscrewing motion. The threaded posts were retained within the internal aspect of the caps upon removal. The final prosthesis was secured to the SRAs using prosthetic screws torqued to 15 Ncm (Figure 3r,s). This method shortened the chairside time and preserved occlusal anatomy by minimizing the access size; attention to cylinder bonding area and component handling was necessary to prevent detachment or screw-related complications (Figure 3). The entire procedure time is about 30–45 min.

### 2.4. Case 4—Closed-Mouth Pickup Conversion—Example 2 (EasyPro Denture Conversion, Keystone Dental Group)

**Figure 4:**
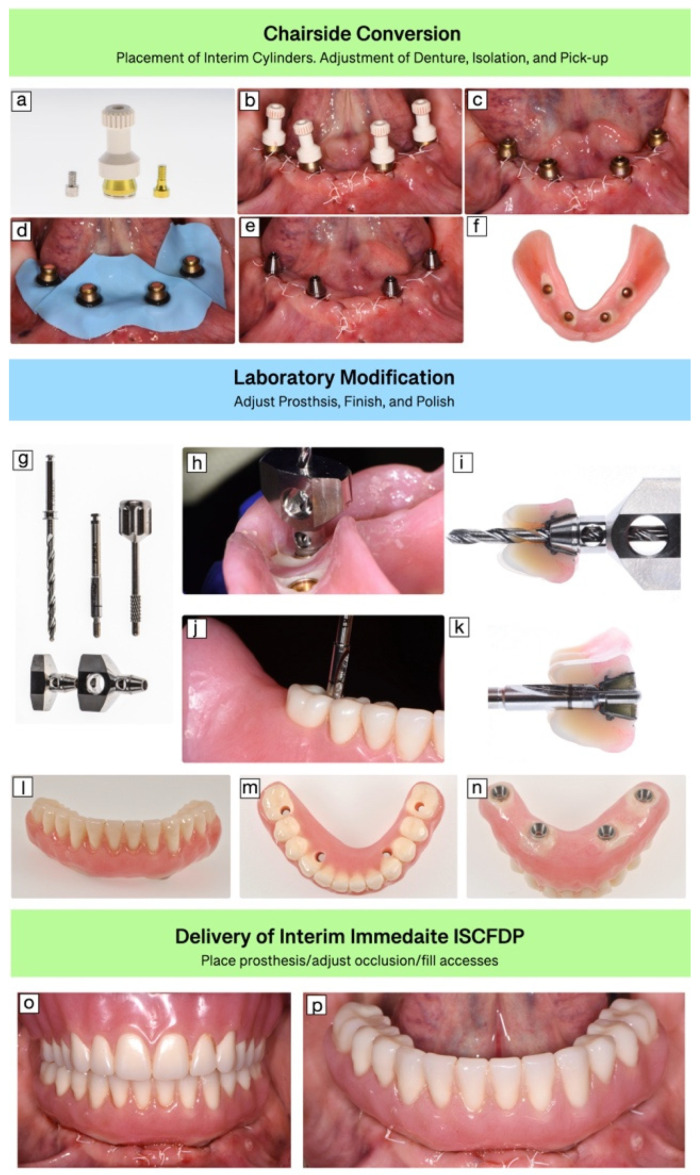
(**a**) Preassembled dual-cylinder units; (**b**) Preassembled dual-cylinder units secured on the abutments using interim titanium screws; (**c**) PEEK handles removed; (**d**) Rubber dam and block-out material in situ; (**e**) Internal silver titanium copings remained on the multi-base abutments; (**f**) External gold titanium copings were retained within the prosthesis; (**g**) Proprietary three-step drill system; (**h**) Drill guide and the pilot drill creating access from the intaglio surface; (**i**) Cross-section view of drill guide and pilot drill going through the prothesis from the intaglio surface; (**j**) Access drill; (**k**) Cross-section view of access drill going through the prothesis from the occlusal surface; (**l**) Finished and polished interim ISCFDP in frontal view; (**m**) Occlusal view; (**n**) Silver titanium copings were removed from the abutments and securely reinserted into the gold titanium copings within the prosthesis; (**o**) Frontal view of the interim immediate-loading ISCFDP; (**p**) Close-up frontal view.

This closed-mouth system employed preassembled dual-cylinder units (external gold titanium coping within the prosthesis; internal silver titanium coping on the abutment) with detachable PEEK handles (Figure 4a). After implant placement with adequate primary stability (insertion torque >35 Ncm) and multi-unit abutment connection (35 Ncm), temporary coping bases and copings were secured to the abutments, and the PEEK handles were removed (Figure 4b,c).

The intaglio surface of a prefabricated mandibular denture was loaded with a quick-setting polyvinyl siloxane (PVS) material and seated in centric occlusion to transfer the abutment positions. Following verification and relief, rubber-dam isolation was established, and polymethyl methacrylate (PMMA) resin was applied to the intaglio surface. The denture was seated intraorally, and the patient was guided into centric occlusion during polymerization to preserve the vertical dimension and occlusal scheme. After approximately 9 min, the prosthesis was removed using a vertical dislodging force; the copings were retained within the prosthesis, while the base components remained attached to the abutments (Figure 4d–f). Any voids were filled with additional PMMA resin, and the provisional restoration was placed in a pressure pot to optimize polymerization at 15 pounds per square inch (PSI) for 5 min.

Final screw access channels were created using a three-step proprietary drill system included in the manufacturer’s starter kit (Figure 4g), as follows:

(1) An intaglio drill was used from the intaglio surface through the TiTemp coping to the occlusal surface of the denture using a drill guide (Figure 4h,i).
(2) An occlusal drill with a built-in stop was then used from the occlusal surface through the pilot channel to the TiTemp coping (Figure 4j,k).
(3) An EasyPro manual reamer was employed to remove any remaining PEEK fragments from the screw access channels, completing the preparation.

The interim ISCFDP was then finished and polished to establish proper anatomical contour and surface smoothness for intraoral delivery (Figure 4l,m). The TiTemp base copings were removed from the MUAs and re-inserted into the TiTemp copings integrated within the ISCFDP with moderate pressure, ensuring a secure fit (Figure 4n). The prosthesis was then definitively secured to the SRAs using prosthetic screws (EasyPro™ Retaining Screw; EasyPro Denture Conversion) torqued to 15 Ncm (Figure 4o,p). This approach preserved the prosthesis structure and occlusion while simplifying access standardization; careful evaluation of coping seating and system-specific steps was essential (Figure 4). The entire procedure time is about 30–45 min.

## 3. Discussions

This article presents four clinical protocols for the conversion of an immediate-loading interim ISCFDP. While each technique utilized distinct components and workflows, all aimed to optimize efficiency, to minimize complications, and to improve mechanical integrity during the immediate loading phase. Conventional conversion methods have long served as the standard of care [6,7,8,9,10]; however, emerging guided and closed-mouth pickup conversion systems offer potential enhancements in prosthesis strength and time efficiency [24,25,26]. Techniques such as stackable guided systems [24], proprietary separable implant screws [25], and proprietary preassembled dual-cylinder units [26] provided unique advantages in specific clinical contexts. Collectively, these methods reflect the growing integration of digital and analog tools in full-arch rehabilitation, underscoring the importance of knowledge and clinician familiarity with evolving technologies. While implant surgery can still be planned using computer-assisted approaches, the conversion may also be performed through an existing interim denture without the need for it to be predesigned or premanufactured, as described in the Case 2. Quantitative clinical parameters, including insertion torque values, polymerization time, and the material properties reported in the literature, were considered to enhance reproducibility and clinical relevance.

### 3.1. Conventional Conversion Technique

**Figure 5:**
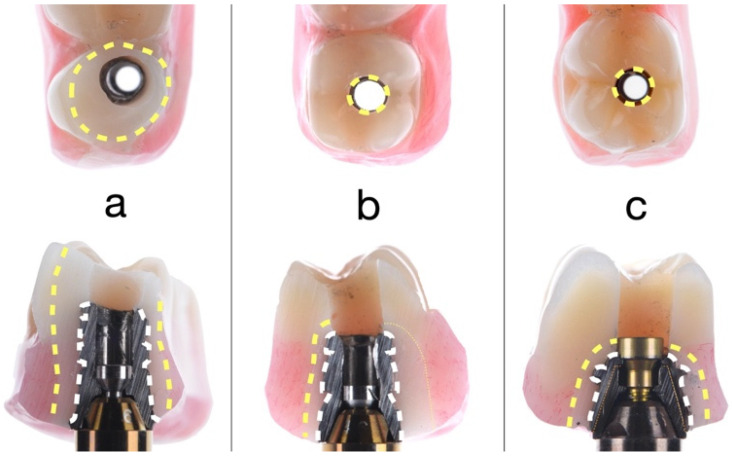
White dotted lines indicate the prosthetic components used in the different conversion techniques, and yellow dotted lines outline the amount of denture relief required during the conversion process. The closed-mouth pickup methods shown in b and c involve less invasive adjustment, preserving the integrity of the denture compared to the conventional method in a: (**a**) Occlusal and cross-sectional views of the conventional conversion technique in Case 1; (**b**) Occlusal and cross-sectional views of the closed-mouth pickup conversion described in Case 3 (Smart Denture Conversion System); (**c**) Occlusal and cross-sectional views of the closed-mouth pickup conversion described in Case 4 (EasyPro Conversion System).

This technique is indicated in situations where cost-effectiveness, workflow flexibility, and availability of conventional components are prioritized, particularly when digital planning or prefabrication is not feasible. Conventional conversion remains the most widely applied protocol because it is cost-effective, accessible, and familiar to most clinicians [1,2,3,4,5,6,28]. However, it is technique-sensitive, requires significant denture modification, and is associated with prosthesis fracture when access openings weaken the acrylic base (Figure 5) [15,16,17].

To address these concerns, some protocols incorporate prefabricated milled dentures [13,23,24] or reinforce the prosthesis with a metal substructure [19,20,21,22]. In addition, because the soft tissue profile may change significantly after bone reduction and implant placement, denture positioning can be challenging. During the conversion process, the block material or interim cylinders often extend through the occlusal plane of the denture, and an opened-mouth technique is used. These factors may result in inaccurate denture positioning during conversion, in turn requiring significant occlusal adjustment of the immediate-loading prosthesis. Table 1 summarizes the advantages and limitations of all four techniques described in this article.

### 3.2. Guided Conversion

This approach is indicated when accurate digital planning and surgical execution can be achieved, particularly in cases requiring improved prosthesis strength, reduced chairside adjustment, and enhanced predictability. Guided conversion integrates digital planning and prefabricated prostheses supported by stackable surgical templates [24,29,30,31,32]. This workflow relies on accurate fixed references; even small deviations can create occlusal discrepancies that necessitate substantial adjustment of the immediate-loading prosthesis. Although interim cylinders must still be picked up intraorally, the prefabricated prosthesis incorporates predesigned access openings based on the virtual implant plan (Figure 2). Compared with conventional chairside pickup, this reduces the extent of denture modification and preserves structural integrity [15,16,17]. An important advantage of this approach is the option to fabricate the prosthesis from high-density, milled monolithic PMMA, which demonstrates approximately 35% greater fracture resistance than conventionally processed denture resin [23]. This contributes to superior structural stability, improved procedural efficiency, and more predictable outcomes. However, this technique is highly dependent on meticulous preoperative planning and precise surgical execution. Minor discrepancies in implant depth, angulation, or rotational positioning—particularly when angled multi-unit abutments are used—can result in prosthesis misalignment and negate the benefits of the guided workflow. In addition, immediate loading requires adequate primary implant stability, which may not always be achievable. When stability is insufficient, clinicians must revert to delivering a conventional complete denture. For this reason, a conventional prosthesis should always be prepared as a contingency. Despite higher costs, longer lead time, and the need for larger access openings due to abutment geometry, guided conversion remains a valuable option when digital infrastructure, surgical accuracy, and prosthodontic expertise are available [24,29].

### 3.3. SMART Denture Conversion

This system is indicated when preservation of occlusal relationships, reduced chairside time, and simplified closed-mouth workflows are desired, especially in patients with an established vertical dimension of occlusion. SMART Denture Conversion employs low-profile titanium cylinders secured with separable screws that disengage during pickup, leaving the PEEK caps and cylinders within the prosthesis while the threaded posts remain on the abutments (Figure 3) [25]. This closed-mouth approach allows the prosthesis to be seated in centric occlusion, preserving the vertical dimension and reducing the need for extensive occlusal adjustments. The smaller access openings also minimize weakening of the denture base compared with conventional techniques [33]. The advantages of this system also include reduced chairside time (approximately one hour) [25]. Moreover, the closed-mouth approach simplifies training and execution, particularly for clinicians familiar with overdenture pickup techniques. It also offers the added advantage of maintaining correct maxillomandibular and occlusal relationships during pickup, which reduces the occlusal errors and adjustment time after conversion. An additional benefit is its intraoperative flexibility: the prefabricated denture can be readily adapted for either immediate loading as an interim ISCFDP or, when primary stability is insufficient, retained as a conventional removable prosthesis without disrupting the treatment workflow.

A limitation of this technique is that it requires careful handling of precoated components to prevent detachment during polymerization. Additionally, screw loosening has been reported more frequently with this system [33], underscoring the importance of thorough verification and follow-up. Furthermore, it relies on proprietary components and tools, which may limit accessibility in certain clinical environments. Despite these considerations, the SDC technique offers a viable and efficient alternative to conventional and guided protocols, particularly in settings where procedural simplicity, time efficiency, and prosthetic integrity are prioritized.

### 3.4. EasyPro Denture Conversion

This technique is indicated in cases requiring simplified handling, reduced technique sensitivity, and improved prosthesis integrity without additional laboratory preparation. EasyPro Conversion uses preassembled dual-cylinder units consisting of external and internal titanium copings. During conversion, the external coping is retained within the prosthesis while the internal coping remains on the abutment, allowing straightforward reassembly. Similar to the SDC system, this system simplifies seating, standardizes screw-access creation with a guided drilling protocol, and maintains occlusal stability through a closed-mouth pickup. Compared with the SMART system, EasyPro eliminates the need for precoating and reduces the risk of component detachment. The EasyPro system also shares the closed-mouth approach’s advantages of simplified training, preservation of occlusal relationships to minimize adjustments, and flexibility to adapt the prefabricated denture for immediate loading or retention as a removable prosthesis without disrupting workflow.

Similar to the SDC technique, this EasyPro method requires careful verification of denture relief and coping seating to prevent pickup misfit. It also depends on proprietary components and tools, which may limit accessibility in some clinical environments. Nonetheless, EasyPro offers an efficient, less technique-sensitive workflow that enhances prosthesis integrity and reduces intraoral adjustment time.

### 3.5. Comparative Analysis of Conventional Conversion Versus Three Conversion Methods

The direct comparative data among the guided conversion, Smart Denture Conversion (SDC), EasyPro, and the conventional denture conversion method remain limited. To date, only one laboratory study has evaluated the SDC protocol against the conventional technique, reporting comparable flexural strength but a substantially higher incidence of prosthetic screw loosening in the SDC group (84%) compared with the conventional technique (4%) [33]. This may reflect differences in the component geometry and screw-retention mechanisms unique to the system. Beyond this, most reports of SDC and EasyPro are confined to clinical technique articles and case presentations with minimal supporting evidence [25,34]. From a clinical perspective, selection of a conversion technique should be based on factors such as available digital workflow, need for occlusal preservation, procedural efficiency, and clinician familiarity. Conventional techniques offer flexibility and guided workflows improve predictability, while closed-mouth systems simplify execution and preserve prosthesis integrity.

The conventional conversion technique remains the most established and predictable method and continues to serve as the clinical standard [1,2,3,4,5,6,28]. Its simplicity and accessibility are offset by drawbacks such as reduced prosthesis strength, risk of fracture from large access openings, and frequent intraoral adjustments [15,16,17]. Guided conversion strategies have been developed to mitigate these shortcomings by improving structural integrity and reducing chairside modification [24,30,31,32]. While promising, their success depends on accurate preoperative planning, precise implant placement, and significant investment in digital and laboratory infrastructure. In parallel, fully digital conversion protocols using photogrammetry or intraoral scanning have gained attention for the immediate loading of ISCFDPs [35,36]. These workflows can eliminate the need for conventional pickup procedures by directly capturing implant positions. Recent studies have further demonstrated the feasibility of fully digital conversion approaches for full-arch implant rehabilitation [37]. However, they require relatively expensive equipment, substantial training for both clinicians and laboratory technicians, and seamless digital integration to be effective. As such, their clinical applicability remains limited in many practice environments. By contrast, closed-mouth systems, such as SDC and EasyPro, represent a conceptual shift from both conventional and guided approaches. These techniques preserve occlusion during pickup, minimize access size, and streamline the clinical workflow. Preliminary reports have suggested they may enhance prosthesis integrity and reduce chairside time. However, their adoption is constrained by proprietary component requirements, limited availability, and a lack of robust in vivo or long-term outcome data.

The interim ISCFDP plays a pivotal role in immediate loading and frequently serves as the reference for subsequent digital scans used in definitive prosthesis fabrication [38]. Therefore, establishing predictable, structurally sound interim prostheses is essential to optimizing fully digital workflows. Future studies are required to validate the long-term performance of guided and closed-mouth conversion systems and to clarify their biological, mechanical, and patient-centered outcomes relative to conventional protocols. Immediate-loading protocols for full-arch implant rehabilitation have demonstrated favorable clinical outcomes, with high survival rates and predictable short-term performance. Recent clinical studies have further supported the reliability of these protocols in fully edentulous patients [39].

## 4. Summary

Despite the inherent challenges of immediate loading in full-arch implant rehabilitation, multiple conversion techniques have been developed to improve their efficiency, predictability, and prosthetic integrity during the interim phase. This clinical report describes and compares four distinct workflows, including conventional denture conversion, guided conversion, Smart Denture Conversion (SDC), and the EasyPro system. Each approach presents unique procedural and mechanical advantages, with specific clinical indications and logistical considerations. By outlining the requirements, benefits, and limitations of each technique, the report provides clinicians with practical guidance for selecting the most appropriate protocol for individual cases. These methods reflect the growing integration of digital and analog tools in full-arch rehabilitation, underscoring the need for the continued refinement of protocols and validation of their long-term clinical performance.

## Figures and Tables

**Table 1:** Requirements, advantages, and disadvantages of the four conversion techniques.

	Conventional Conversion	Guided Conversion	Closed-Mouth Pickup Conversion—SMART Denture Conversion (SDC)	Closed-Mouth Pickup Conversion—EasyPro System
Requirement	Existing denture Interim cylinders	s-CAIS planning Prefabricated immediate-loading prosthesis Interim cylinders	Existing denture Proprietary SDC system, including instruments and prosthetic components	Existing denture Proprietary EasyPro system, including instruments and prosthetic components
Advantages	Well-established Lower cost Accessible to clinicians Flexible workflow	Well-established Preserves prosthesis integrity Shorter chairside procedure Predictable outcome through digital planning	Preserves denture integrity leading to a stronger prosthesis and more intact occlusal anatomy Shorter chairside time Flexible workflow	Preserves denture integrity leading to a stronger prosthesis and more intact occlusal anatomy Shorter chairside time Flexible workflow
Disadvantages	Technique-sensitive Extensive denture adjustment needed to accommodate interim cylinders Risk of prosthesis fracture Loss of occlusal anatomy of the prosthesis Opened-mouth pickup may cause occlusal discrepancies requiring major adjustment of the immediate-loading prosthesis Longer chairside procedure	Requiring advanced knowledge of the digital workflow High planning and manufacturing cost Requires precise surgical execution following digital plan Low intraoperative flexibility Accurate fixed references are essential, as errors can cause occlusal discrepancies requiring major adjustment of the immediate-loading prosthesis Conventional denture may be required as a contingency	May not be available in some markets Additional laboratory preparation for precoating interim cylinders Small bonding surface on the interim cylinders leading to detachment Additional cost to purchase the proprietary instruments and prosthetic components Requires system-specific training and knowledge Limited clinical evidence and prosthetic screw loosening was reported	May not be available in some markets Seating between internal and external copings should be carefully evaluated Additional cost to purchase the proprietary instruments and prosthetic components Require system-specific training and knowledge Limited clinical evidence

Note: s-CAIS: static computer-aided implant surgery.

## Data Availability

Data supporting the findings of this study are available from the corresponding author upon reasonable request. The data are not publicly available due to patient privacy considerations.
